# A nomogram prediction of coronary artery dilation in Kawasaki diseases based on mtDNA copy number

**DOI:** 10.3389/fimmu.2024.1448558

**Published:** 2024-08-14

**Authors:** Mou Peng, Peng Yue, Yue Zhang, Hong Li, Yimin Hua, Yifei Li, Hong Zheng, Fangfei Liu

**Affiliations:** ^1^ Key Laboratory of Birth Defects and Related Diseases of Women and Children of Ministry of Education (MOE), Department of Pediatrics, West China Second University Hospital, Sichuan University, Chengdu, Sichuan, China; ^2^ Department of Nursing, West China Second University Hospital, Sichuan University, Chengdu, China

**Keywords:** Kawasaki disease, mitochondrial DNA copy number, nomogram prediction, risk factor, clinical outcome

## Abstract

**Objective:**

The level of mitochondrial DNA copy number (mtDNA-CN) in peripheral blood cells had been identified to be involved in several immune and cardiovascular diseases. Thus, the aim of this study is to evaluate the levels of mtDNA-CN in Kawasaki disease (KD) and to construct a nomogram prediction for coronary artery lesions in children with KD.

**Methods:**

One hundred and forty-four children with KD diagnosed from March 2020 to March 2022 were involved in the study. The clinical features and laboratory test parameters of these children were assessed between the KD and normal groups. Univariable and multivariable analyses were performed sequentially to identify the essential risk factors. Subsequently, a nomogram prediction was constructed.

**Results:**

A total of 274 children were included in the analysis. Of these, 144 (52.6%) represented the KD group. Peripheral blood DNA mtDNA qPCR showed that the -log value of mtDNA-CN in the KD group (6.67 ± 0.34) was significantly higher than that in the healthy group (6.40 ± 0.18) (P<0.001). The area under the ROC curve for mtDNA-CN in distinguishing KD was 0.757. MtDNA-CN (OR = 13.203, P = 0.009, 95% CI 1.888–92.305), RBC (OR = 5.135, P = 0.014, 95% CI 1.394–18.919), and PA (OR = 0.959, P = 0.014, 95% CI 0.927–0.991) were identified as independent risk factors for coronary artery dilation in children with KD. Finally, the nomogram predictive was established based on the results of multivariable analysis, demonstrating the satisfied prediction and calibration values.

**Conclusion:**

The results of this study revealed that mtDNA-CN could be used as a biomarker in predicting the development of KD. Furthermore, the higher the mtDNA-CN was significantly associated with coronary artery dilation in KD.

## Introduction

Kawasaki disease (KD), also known as mucocutaneous lymph node syndrome (MCLS), is an acute febrile rash disease in children characterized primarily by systemic vasculitis, which is a kind of disease with immune attacks to multiple organs (1). In recent years, the incidence of KD has increased, making it one of the leading causes of acquired heart disease in children (2–5). The most serious complication associated with KD is coronary artery injury, which includes coronary artery dilatation (CAD), stenosis, aneurysm, and even myocardial infarction. Current treatment for KD is highly effective, with intravenous immunoglobulin (IVIG) being one of the most cost-effective medical treatments available, providing significant short- and long-term cost savings (1). The combination of aspirin and IVIG shows good efficacy in most acute cases, although achieving effective results with IVIG remains challenging in refractory cases. Diagnosing KD is relatively straightforward due to its distinct clinical manifestations and specific laboratory tests. However, the current challenge lies in assessing the risk of coronary artery disease in affected patients. Consequently, there is an urgent need for tools to predict coronary artery disease in children with KD, enabling personalized risk-based management.

Mitochondria, often referred to as the powerhouses of the cell, are crucial for energy production, cellular metabolism, and various physiological processes (6, 7). Mitochondria contain their own genetic material, mitochondrial DNA (mtDNA), a double-stranded molecule encoding 2 ribosomal RNAs, 22 transfer RNAs, and 13 polypeptides of the respiratory chain (7). Each cell contains between 100 to 1000 mitochondria, and each mitochondrion houses 2 to 10 copies of mtDNA, making it a multicopy genome (8). Unlike nuclear DNA, mtDNA is particularly susceptible to damage due to the lack of histone protection and effective DNA repair mechanisms (9). Under various stress conditions, including trauma, autoimmune diseases, HIV, diabetes, cardiovascular disorders, systemic lupus erythematosus (SLE), amyotrophic lateral sclerosis (ALS), psychological stress, and various cancers, mtDNA can be released into circulation (7, 10–15). Previous studies have highlighted the significant role of mtDNA-CN in various cardiovascular and immune-related diseases. The researchers found that reduced mtDNA-CN in peripheral blood is significantly associated with the incidence of cardiovascular disease, even after adjusting for traditional risk factors such as age, gender, and smoking status. Similar associations have been found between decreased mtDNA-CN and heart failure. Highlighting the potential of mtDNA-CN as a biomarker for heart failure. Hypertension has also been associated with reduced mtDNA-CN. The mitochondrial dysfunction indicated by lower mtDNA-CN may contribute to the pathogenesis of hypertension through impaired vascular smooth muscle function and increased oxidative stress. The researchers also found that oxidized mtDNA induces gasdermin D oligomerization in Systemic Lupus Erythematosus, highlighting its role in the inflammatory process suggesting the potential of mtDNA-CN as a biomarker for Rheumatoid Arthritis. Recent advancements in molecular biology techniques have enabled researchers to explore mtDNA-CN as a potential clinical laboratory indicator. Increasing evidence suggests that mtDNA can serve as a biomarker for predicting the occurrence and progression of numerous diseases (16, 17).

Herein, we carried out this research to illustrate the changes of mtDNA-CN in peripheral blood cells between healthy participants and KD patients. Then we attempted to identify the potential role of mtDNA-CN in assessing the persistent CAD in middle-term follow-up of KD, which revealing the advantages of mtDNA-CN in KD management compared with general laboratory parameters.

## Methods

### Ethics statement

This study was conducted following the ethical guidelines and was approved by the Ethics Committee of West China Second University Hospital, Sichuan University (approval number: 2021–069). Informed consent was obtained from all parents or legal guardians of the patients, who provided their consent for including their child’s clinical and imaging details in the manuscript for publication purposes. This is a prospectively designed research. There were two groups involved in the observational cohort, which were KD patients (KD group) and healthy population (HT group).

### Patients

This was a prospective, single-center, observational study that enrolled children with KD between 2020 and 2022 at West China Second University Hospital, Sichuan University. The study conformed to the principles of the Declaration of Helsinki and was approved by the Ethics Committee of West China Second University Hospital, Sichuan University. Two pretrained physicians collected the data, and all clinical data were verified using electronic medical records.

### Inclusion and exclusion criteria

The inclusion criteria of KD group included the followings: 1) the diagnosis of KD referred to the criteria in “Diagnosis, Treatment, and Long-Term Management of KD: A Scientific Statement for Health Professionals From the American Heart Association” (1); 2) identification of KD with CAD either by transthoracic echocardiography, CT angiography or transcatheter angiography and identification of CAD onset within the acute or subacute phase of KD, which were persistent at the end of the 1 year follow-up; 3) the age of enrolled patients ranged from 0 to 12 years; 4) the peripheral blood samples should be collected at the time of initial hospital admission before IVIG administration; 5) well-collected and completed programmed questionnaires, basic essential information, clinical manifestation, hematological examination results, therapeutic procedure, echocardiography results, and follow-up outcomes.

While, the exclusion criteria of KD group included the followings: 1) patients with definite infectious disease at the same time with KD; 2) patients with birth defects or cardiovascular malformations history; 4) patients with autoimmune disease prior to KD onset; 5) treatment with anticoagulant or antiplatelet medication prior to KD onset; 6) patients with cardiac surgeries history; 7) suspicion of myocarditis prior to KD onset; 8) use of glucocorticoids prior to IVIG administration; 9) use of monoclonal antibodies, including tumor necrosis factor (TNF)-α and interleukin (IL)-6 antibodies; 10) incomplete medical archive or laboratory tests; 11) lost follow-up; 12) family history of coronary artery diseases.

Moreover, the volunteers of HT group were included among the healthy population with age matched who received general body examinations, including routine blood test, hepatic and renal function, coagulation function, echocardiography, chest X-ray and electrocardiogram. Also, the cases had been excluded from HT group due to previous heart attacks, congenital malformation of coronary artery and heart, past history of autoimmune diseases, any open chest or heart surgery, recent (<1 month) respiratory infection and incomplete medical archive.

The observational end point of the cohort had been designed as middle-term follow-up of one year after hospital discharge or patient death.

### Blood samples collection and nucleotide extraction

A total of 4ml of peripheral blood samples were collected from the patients on admission, of which 2ml was stored in EDTA tubes, then such samples placed in a -40°C refrigerator. The experimental samples were kept and registered by special personnel, and the extraction time of DNA shall not exceed 6 months. Blood DNA Kit (D3392–02) was used to extract DNA from peripheral blood samples, and the concentration and purity of DNA was determined and stored at -20°C.

### MtDNA-CN

To obtain the DNA sequence amplification of mt-ND1, design Primers (Forward ATACCCATGGCCAACCTCCTA; Reverse TAGGTTTGAGGGGGAATGCTG) were used to amplify the whole mitochondrial mt-ND1 gene to quantify the mitochondrial genome. Fragments were amplified using NEB Q5 High-Fidelity DNA polymerase. Then mt-ND1 sequence was connected to the T vector according to the Mighty TA-cloning Reagent Set for Prime STAR^®^ (Code N0.6019 TaKaRa Company) kit instructions for research and linearize the plasmid and make a gradient concentration standard. Calculate the number of DNA copies according to the following formula:

DNA (copies/ul) = (DNA Concentration (ng/ul) × 6.022×10^23^)/(Length(bp)×1×10^9^×660) (6.022×10^23^= Avogadro’s constant, 1×10^9^= mole amount converted to ng, 660= average mass of a pair of double-stranded DNA bases (g), Length=vector+length of insert). Dilute the linearized plasmid 10-fold with ddH2O to a concentration of 10^8^-10^3^ copies/ul to make a standard.

### Statistical analysis

Univariable analysis was conducted using IBM SPSS 26.0 (IBM SPSS Inc., Chicago, IL, USA). Quantitative data are presented as means and standard deviations (SDs), while qualitative data are expressed as numbers of individuals. Differences between two groups were assessed using independent t-tests or Mann–Whitney U tests for continuous variables, and the χ² test or Fisher’s exact test for categorical variables. Multivariable logistic regression analyses, nomograms, and model evaluations were formulated and conducted using R (version 4.1.2) within the RStudio platform. P values < 0.05 were considered statistically significant.

## Results

### Characters of included patients

One hundred and ninety-two patients were initially enrolled in the KD group. Eighteen patients were subsequently excluded due to lost follow-ups, 11 patients who presented CAD in the acute or subacute phase but recovered at the initial follow-up end point (1 year), 5 patients were identified as having congenital disorders, 5 had concomitant injuries to other systems,3 patients had been provided glucocorticoids before IVIG, and 6 patients did not complete coagulation tests. Thus, the final analysis included 144 patients in the KD group. Among the KD group, 112 cases were absent from any CAD, while 32 patients presented persistent CAD at the end of 1-year follow-up. In the HT group, 108 volunteers were initially included. Among them, 7 participants had congenital birth defects, 6 participants suffered autoimmune diseases, 13 participants were diagnosed with respiratory infection in the last 1 month, 3 participants reported positive family history of heart attacks. Consequently, 79 healthy participants with complete medical records were included for further analysis. Baseline information was compared between the two groups, revealing no significant differences in gender distribution, average age, or body weight.

### To evaluate the level of mtDNA-CN in peripheral blood between KD and HT

**Figure 1:**
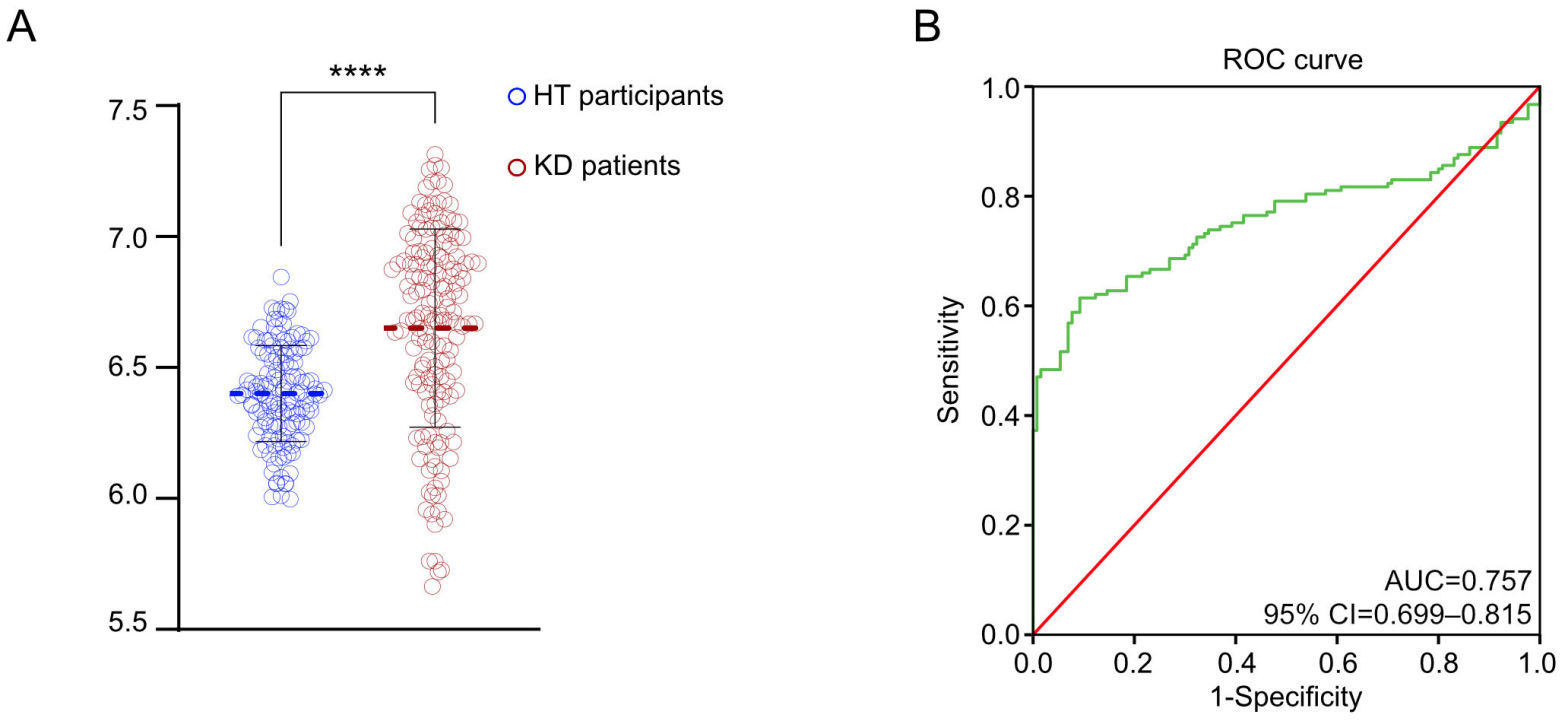
Elevated mtDNA-CN in KD patients. **(A)** The violin plot demonstrated the different measured level of mtDNA-CN; **(B)** The ROC curve in distinguishing the different KD patients. ****P value < 0.0001.

The level of mtDNA-CN detected in the peripheral blood of the KD group (10^6.67 ± 0.34 copies/ul) was significantly higher than that of the HT group (10^6.40 ± 0.18 copies/ul), with a statistically significant difference (P<0.001) (**Figure 1A**). To further assess the value of mtDNA-CN in identifying KD, ROC analysis had been enrolled to evaluate its predictive contribution (**Figure 1B**). According to the ROC plot, a sufficient role of mtDNA-CN in distinguishing KD had been identified with an AUC of 0.757 (95% CI 0.699–0.815).

### Univariate analyses of clinical characteristics in KD with and without CAD

A total of 144 eligible patients who were diagnosed KD were enrolled in further subgroup analysis. There were 112 CAD-free KD patients, and 32 cases had been identified as persistent CAD at the end of 1-year follow-up. Then, the univariable analysis had been completed among all the documented clinical parameters and the assessed mtDNA-CN. Accordingly, several indicators were revealed to be significantly associated with persistent CAD in KD, including body mass index (BMI, p=0.016), mtDNA-CN (p=0.001), red blood cell (RBC, p=0.044), platelets (PLT, p=0.024), procalcitonin (PCT, p=0.002), γGT (p=0.002), prealbumin (PA, p=0.007), alkaline phosphatase (ALP, p=0.015), and uric acid (UA, p=0.000) (**Table 1**).

**Table 1:** Univariate analysis for risk factors.

Variables	KD with non-CAD (n=112)	KD with persistent CAD (n=32)	P value
Fever duration (days)	5.41 ± 2.640	4.90 ± 2.72	0.338
Age (years)	3.20 ± 1.95	3.19 ± 2.68	0.985
BMI (kg/m^2^)	15.52 ± 1.51	16.27 ± 1.59	0.016*
mtDNA-CN (log (copies/ul))	6.47 ± 0.36	6.71 ± 0.30	0.001*
Gender			0.222
Male	60 (53.6%)	21 (65.6%)	
Female	52 (46.4%)	11 (34.4%)	
Ethnic			0.571
Minorities	4 (3.6%)	2 (1.3%)	
Han Nationality	108 (96.4%)	30 (98.7%)	
WBC (×10^9^/L)	13.80 ± 4.77	13.41 ± 5.78	0.700
N (%)	69.66 ± 14.51	64.02 ± 20.25	0.080
L (%)	21.44 ± 11.23	25.84 ± 15.58	0.077
M (%)	6.91 ± 6.04	5.68 ± 2.59	0.264
RBC (×10^12^/L)	4.15 ± 0.38	4.45 ± 0.85	0.044*
HGB (g/L)	111.86 ± 11.20	111.94 ± 11.94	0.972
PLT (×10^9^/L)	351.44 ± 109.98	303.34 ± 87.32	0.024*
HCT (%)	33.33 ± 2.96	33.85 ± 2.99	0.380
PCT (%)	0.35 ± 0.09	0.29 ± .07	0.002*
CRP (mg/L)	75.87 ± 45.27	93.93 ± 52.21	0.057
ESR (mm/h)	65.87 ± 25.57	60.91 ± 31.11	0.359
ALT (U/L)	80.48 ± 135.52	124.97 ± 237.24	0.176
AST (U/L)	75.38 ± 141.70	106.88 ± 233.41	0.346
AST/ALT	1.41 ± 0.78	1.17 ± 0.70	0.111
TB (mmol/L)	12.21 ± 18.40	12.11 ± 12.63	0.977
DBIL (mmol/L)	6.49 ± 14.62	6.63 ± 10.54	0.961
IDIL (mmol/L)	4.93 ± 2.94	5.42 ± 3.20	0.418
ALB (g/L)	38.99 ± 4.44	38.34 ± 5.10	0.483
GLB (g/L)	27.30 ± 20.57	25.52 ± 7.89	0.632
γGT (U/L)	54.85 ± 63.75	113.80 ± 153.48	0.002*
LDH (U/L)	309.05 ± 143.42	293.90 ± 123.99	0.589
PA (mg/L)	61.78 ± 22.41	50.16 ± 15.19	0.007*
ALP(U/L)	197.13 ± 64.22	234.28 ± 106.81	0.015*
UN (mmol/L)	3.22 ± 1.10	3.71 ± 1.81	0.057
Cr (umol/L)	26.99 ± 6.21	27.25 ± 9.79	0.858
CYSC (mg/L)	0.79 ± 0.15	0.83 ± 0.18	0.148
UA (umol/L)	210.31 ± 64.82	263.81 ± 79.45	0.000*
IVIG resistance (n)	38 (33.9%)	8 (25%)	0.325
ECG Abnormal (n)	16 (16.2%)	7 (24.1%)	0.375
Days of aspirin administration	5.4198 ± 1.65204	5.5161 ± 2.75525	0.809
Aspirin dose			0.734
Low dose	6 (5.4%)	2 (6.3%)	
High dose	106 (94.6%)	30 (93.8%)	

*statistically significant difference; BMI, body mass index; mtDNA-CN, mitochondrial DNA copy Number; N, neutrophil; L, lymphocyte; M, monocyte; HGB, hemoglobin; PCT, procalcitonin; γGT, γ glutamyltransferase; PA, serum prealbumin; UA, uric acid; IVIG, intravenous immunoglobulin; ECG, electrocardiogram; KD, kawasaki disease; CAD, Coronary Artery Disease; continues data were presented as mean ± SD; categorical variables were presented as percentage.

### MtDNA-CN serves as an independent predictor for persistent CAD in KD

Based on the univariable analysis, the variables of BMI, mtDNA-CN, RBC, PLT, PCT, γGT, PA, ALP, and UA had been taken into the multivariable logistic regression analysis. However, only three parameters maintain their significance in predicting persistent CAD. Thus, the multivariable logistic regression analysis demonstrated mtDNA-CN (OR=13.203, p=0.009, 95%CI 1.888–92.305), RBC (OR=5.135, p=0.014, 95%CI 1.394–18.919), and PA (OR=0.959, p=0.014, 95%CI 0.927–0.991) as independent factor to distinguish persistent CAD among KD patients (**Table 2**).

**Table 2:** Multivariable analysis for risk factors for KD with persistent CAD.

Features	B	SEM	OR (95% CI)	P-value
BMI	0.339	0.187	1.404 (0.973, 2.026)	0.070
mtDNA-CN	2.580	0.992	13.203 (1.888, 92.305)	0.009 *
RBC	1.636	0.665	5.135 (1.394, 18.919)	0.014 *
PLT	-0.002	0.008	0.998 (0.982, 1.015)	0.822
PCT	-5.628	9.515	0.004 (0.000, 451275.069)	0.554
γGT	0.005	0.003	1.005 (0.999, 1.011)	0.110
PA	-0.042	0.017	0.959 (0.927, 0.991)	0.014 *
ALP	0.002	0.005	1.002 (0.992, 1.011)	0.743
UA	0.006	0.004	1.006 (0.998, 1.013)	0.148

*statistically significant difference; BMI, body mass index; mtDNA-CN, mitochondrial DNA copy number; PCT, procalcitonin; γGT, γ glutamyltransferase; PA, serum prealbumin; UA, uric acid; KD, kawasaki disease; CAD, coronary artery dilation.

### Establishing a nomogram prediction for persistent CAD in KD patients using mtDNA-CN

**Figure 2:**
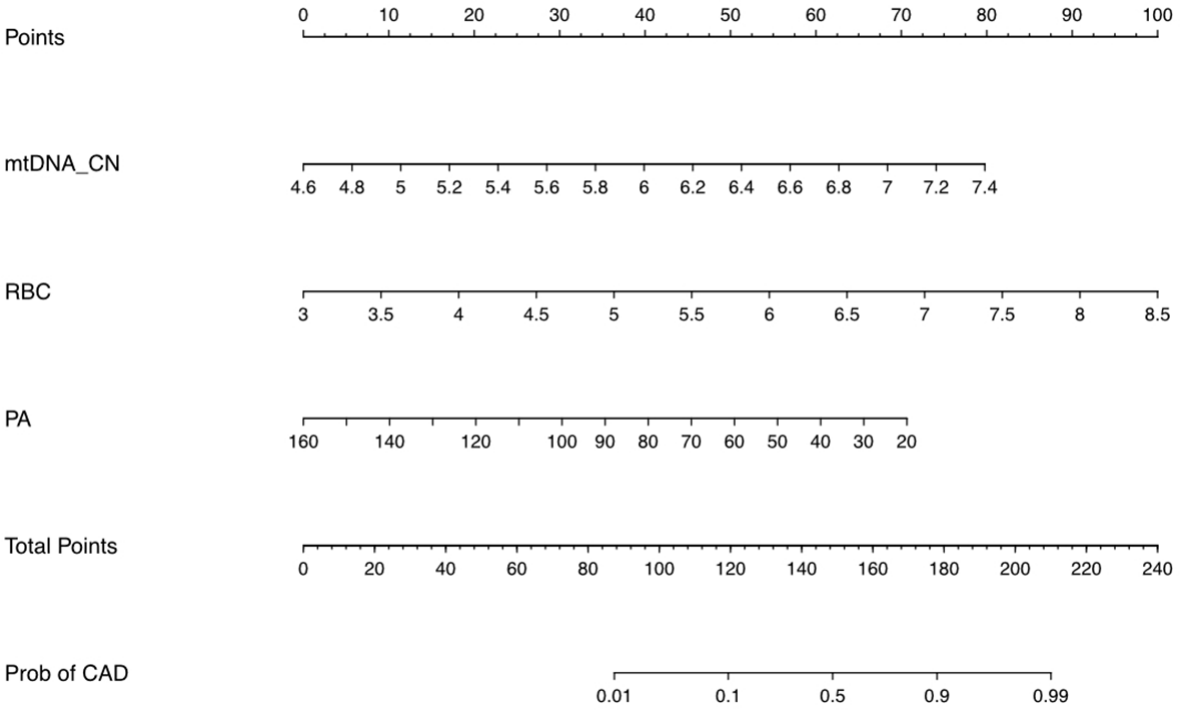
The nomogram prediction scores based on multivariable analyses. The value of each variable was assigned a score on the point scale axis. A total score could be calculated by adding each single score totally. By projecting the total score to the lower total point scale, we were able to estimate the probability of persistent CAD in children with KD.

**Figure 3:**
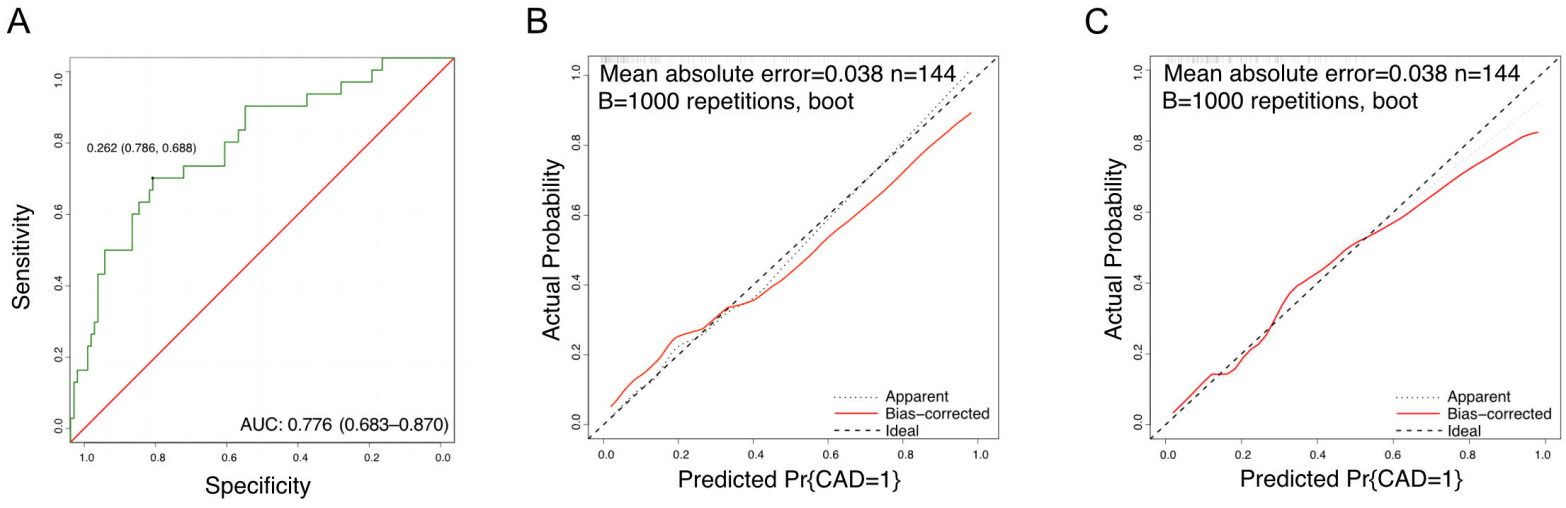
The prediction value of nomogram model. **(A)** The ROC curves of the nomogram scores; **(B)** Training calibration plot analyses for nomogram scores; **(C)** Validation calibration plot analyses for nomogram scores.

The identified independent risk factors for persistent CAD in KD were enrolled to establish a nomogram prediction model, presenting in the **Figure 2**. The higher cumulative points, which should be calculated by summing the assigned scores for each predictor in the nomogram system, were tightly associated with an definitely increased risk of KD with persistent CAD. Based on the ROC analysis, the nomogram prediction model showed an efficient prediction, with an AUC of 0.776 (95%CI 0.683–0.870) (**Figure 3A**). Calibration curves were plotted to evaluate the predictive accuracy of the nomogram. **Figures 3B, C** demonstrated the consistency of the prediction and actual observation in both the training and validation cohorts, indicating the perfect calibration ability of this predictive nomogram.

## Discussion

The gene content of vertebrate mtDNA has remained stable throughout evolution, although the gene order may vary. The mtDNA-CN in a single somatic cell in humans ranges from 10^3 to 10^4, with tissue-specific variations (6). The mtDNA-CN correlates with both physiological and pathological states of the body. Numerous studies have elucidated the intricate relationship between altered mtDNA-CN and various diseases (18, 19). Elevated mtDNA-CN has been associated with conditions characterized by increased energy demands, such as cancer and obesity (7). Conversely, reduced mtDNA-CN has been observed in neurodegenerative disorders like Alzheimer’s and Parkinson’s diseases, as well as in metabolic disorders like diabetes (10). A recent study conducted by Johns Hopkins University found that the number of mitochondrial DNA copies in peripheral white blood cells can predict the risk of cardiovascular disease. This finding enhances risk classification and informs the timing of statin use for prevention. The study analyzed data from a cohort of 21,870 patients (including 20,163 baseline controls without cardiovascular disease) over an average follow-up period of 13.5 years, demonstrating that a lower mtDNA-CN is associated with an increased risk of cardiovascular disease (20). The risk of cardiovascular disease escalates with decreasing mtDNA-CN. Even after adjusting for traditional risk factors such as gender, blood lipid levels, blood pressure, smoking, and hypertension medication, mtDNA-CN remained significantly associated with cardiovascular disease incidence, suggesting that mtDNA-CN is an independent risk factor (20). These findings propose that mtDNA-CN could serve as a valuable biomarker for disease risk assessment, diagnosis, and prognosis. When compared to other commonly used biomarkers for KD, such as C-reactive protein (CRP) and erythrocyte sedimentation rate (ESR), mtDNA-CN shows comparable or even superior predictive accuracy. CRP and ESR are traditional inflammatory markers frequently used in the diagnosis of KD and other inflammatory conditions. The AUC values for CRP and ESR typically range from 0.65 to 0.80. While these markers are useful, their predictive accuracy can be limited by various factors, including the presence of other inflammatory conditions that can elevate CRP and ESR levels. Alterations in mtDNA-CN reflect mitochondrial dysfunction, which is increasingly recognized as a key factor in the pathophysiology of inflammatory diseases, including KD. The mtDNA-CN alterations may occur earlier in the disease process compared to changes in CRP and ESR. This early detection capability is crucial for timely intervention and management of KD, potentially improving patient outcomes.

KD is a vascular inflammatory condition affecting multiple systems and organs. CAD associated with KD has become the leading cause of acquired heart disease in developed countries and many regions of China (3, 5). A giant coronary artery aneurysm is a major risk factor for coronary events in KD, potentially leading to coronary artery stenosis, thromboembolism, myocardial ischemia, and even death (21). Orenstein et al. identified three different but interrelated pathological processes in the progression of coronary artery lesions in children of KD as acute self-limited necrotizing arteritis, subacute/chronic vasculitis, and luminal myofibroblast proliferation (22). Necrotizing arteritis, mediated by neutrophils, is a self-limiting process that starts and ends within the first two weeks of fever. It originates from the endothelium of the coronary artery, progressively damaging the intima, internal elastic layer, media, and external elastic layer, leading to the formation of coronary artery aneurysms (CAA). This process can extend peripherally, causing rupture or thrombosis of CAA within one month. Therefore, anti-inflammatory treatment during the acute phase of KD is crucial to controlling the progression of coronary artery lesions (4). However, current research lacks predictive indicators with high specificity and sensitivity, which significantly limits the early prevention of severe complications of KD. In our study, we found that mtDNA-CN was significantly increased in children with KD compared to healthy individuals, and ROC analysis suggested a high predictive value. Additionally, we conducted statistical analyses to predict the risk of CAD in children with KD based on mitochondrial DNA copies. In single-factor analysis to determine risk factors, we performed multivariable regression analysis for indicators with significant results. We found that mtDNA-CN, RBC count, and PA levels can be used as independent risk factors to evaluate the risk of coronary artery ectasia in children with KD. Standard treatments for KD, such as intravenous immunoglobulin (IVIG) and aspirin, might influence mitochondrial DNA copy number (mtDNA-CN) levels by modulating the inflammatory response and mitochondrial function. IVIG is known for its potent anti-inflammatory and immunomodulatory effects, which could potentially reduce mitochondrial stress and stabilize mtDNA-CN levels. Similarly, aspirin, which serves as both an anti-inflammatory and antiplatelet agent, might also contribute to the stabilization of mtDNA-CN. Currently, there is limited direct evidence specifically linking changes in mtDNA-CN to the treatment responses of IVIG and aspirin in KD.

The association between higher mtDNA-CN and CAD in KD could be attributed to the increased energy demands and mitochondrial biogenesis that occur in response to inflammatory and oxidative stress. Mitochondria are essential organelles that play a critical role in cellular metabolism, energy production, and immune responses. Their dysfunction can significantly contribute to endothelial damage and vascular inflammation, which are key factors in the development of CAD. During KD, the intense inflammatory response generates a significant amount of ROS. This oxidative stress can damage cellular components, including lipids, proteins, and DNA. To counteract this, cells up-regulate mitochondrial biogenesis, resulting in higher mtDNA-CN. Studies have shown that mitochondrial biogenesis is a critical adaptive mechanism in response to oxidative stress, helping to maintain cellular function and viability (6). On the other hand; Mitochondria generate ATP through oxidative phosphorylation, which is crucial for energy-intensive processes during inflammation. The increased mtDNA-CN in KD suggests enhanced mitochondrial activity to meet the elevated energy demands of immune cells engaged in combating the disease (7, 8). Next, mitochondria are involved in the regulation of innate and adaptive immune responses. They influence the activation and proliferation of immune cells, the production of cytokines, and the regulation of apoptosis. Higher mtDNA-CN may reflect the heightened activity and proliferation of immune cells in response to KD, contributing to the inflammatory milieu (10). In addition to this; Mitochondrial damage and the release of mtDAMPs can trigger inflammation through the activation of pattern recognition receptors such as Toll-like receptors (TLRs) and the NLRP3 inflammasome. This inflammatory cascade can result in endothelial cell activation and damage, promoting the development of CAD in KD patients (9, 14). Finally; Vascular inflammation in KD is characterized by the infiltration of immune cells into the vessel walls, leading to endothelial dysfunction and vascular injury. The increased mtDNA-CN may exacerbate this process by enhancing the inflammatory response and contributing to oxidative stress, thereby promoting the progression of CAD (19).

Our findings have significant implications for the early prevention of CAD in KD. For children identified by our prediction model as high-risk, early intervention with IVIG combined with corticosteroids may help prevent the progression of coronary artery complications and achieve the goal of averting CAD. This approach holds profound clinical significance for the prognosis of children diagnosed with KD. Early identification of high-risk patients and the implementation of targeted therapies can potentially reduce the incidence and severity of coronary artery abnormalities, thereby improving the long-term outcomes for KD patients (23). Integrating our nomogram model into clinical practice may enable healthcare providers to personalize treatment approaches and optimize patient care. We believe that our study contributes valuable insights into risk assessment and early intervention strategies for KD, ultimately aiming to improve patient outcomes and reduce the burden of coronary artery complications.

Despite the promising potential of mtDNA-CN as a clinical indicator, several challenges need to be addressed. Standardizing methodologies for measuring mtDNA-CN is crucial to ensure consistency and comparability across different studies and laboratories. Additionally, the influence of confounding factors, such as heteroplasmy (the presence of multiple mtDNA variants), must be carefully considered (24). Future advancements in high-throughput sequencing technologies and computational analysis tools are likely to enhance our understanding of mtDNA-CN quality control. Integrating mtDNA-CN measurements with other omics data, such as transcriptomics and proteomics, could provide a more comprehensive application for assessing mitochondrial function and its associations with cardiovascular diseases (25, 26).

## Conclusion

This study suggested that the elevated mtDNA-CN level was associated with KD. Furthermore, the assessment of mtDNA-CN in peripheral blood cells would serve as an independent parameter in predicting CAD and related complications. This research expanded the application of mitochondrial function analysis in demonstrating clinical prognosis in specific diseases and emphasized the molecular function of mtDNA quality control in cardiovascular diseases.

## Data Availability

The data presented in the study are depository in the OSF database, and accession link was https://osf.io/j39zf/?view_only=e137e34fcb594402a669b661d1877b65.
